# Unilateral RS3PE in a Patient of Seronegative Rheumatoid Arthritis

**DOI:** 10.1155/2013/923797

**Published:** 2013-04-10

**Authors:** Ankur Nandan Varshney, Nilesh Kumar, Ashutosh Tiwari, Ravi Anand, Sashi Ranjan Prasad, Arvind Anand, Abhinandan Mishra, N. K. Singh

**Affiliations:** Division of Rheumatology, Department of General Medicine, Institute of Medical Sciences, Banaras Hindu University, Varanasi 221005, India

## Abstract

Remitting seronegative symmetrical synovitis with pitting edema (RS3PE) is a rare but well-reported clinical entity. It is classically described as symmetrical involvement of both upper extremities. Asymmetrical involvement had also been reported, but unilateral presentation is very rare. We hereby report a case of unilateral RS3PE in a patient of seronegative rheumatoid arthritis which was initially misdiagnosed as cellulitis and was given high dose antibiotics without any significant improvement. Later a rheumatologic consultation leads to a prompt diagnosis, and treatment with steroids leads to dramatic reversal of symptoms. This case demonstrates the rare presentation of this rare clinical entity and highlights the necessity of awareness regarding unilateral disease to clinicians.

## 1. Introduction

Ever since remitting seronegative symmetrical synovitis with pitting edema was described by McCarty et al. [1] in 1985 as a subset of elderly onset rheumatoid arthritis, it has always drawn attention of rheumatologists with its distinct and varied clinical features. RS3PE is characterized by acute onset symmetrical polyarthritis with dramatic onset of pitting edema with extreme tenderness. The other peculiar features include male predominance, old age, negative rheumatoid factor, absence of bony erosions on radiographs, good response to low-dose steroids, and long-term remission after withdrawal of steroids. In all ten original cases described by McCarty the disease was bilaterally symmetrical. Since McCarty's original description over 150 cases of RS3PE has been reported [2]. In almost all the cases it is described as a symmetrical disease involving both hands and rarely the feet [3]. Thus symmetrical presentation is considered as one of the hallmark of disease. However exceptions are always there. RS3PE too presents in an asymmetrical and unilateral pattern, though it is extremely rare. Thus diagnosing it always poses a clinical challenge, and correct diagnosis is delayed often.

We hereby report a case of seronegative rheumatoid arthritis patient who developed acute onset polyarthritis with dramatic onset of pitting edema in left hand and was misdiagnosed to have cellulitis initially, but later a diagnosis of RS3PE was made, and she improved after a course of low-dose prednisolone.

## 2. Case Report

A 68-year-old female, housewife, suffering from multiple peripheral joint pain along with morning stiffness was diagnosed having seronegative rheumatoid arthritis two years back and was given methotrexate (15 mg weekly) and leflunomide (10 mg daily) as per local orthopedics consultation. Her symptoms were well controlled with the medication. Five weeks back, she developed pain in metacarpophalangeal joints and wrist of left side along with swelling of hand. It was acute in onset and moderate-to-severe in intensity to an extent that she was unable to make a fist. There was no radiation of pain. It was associated with morning stiffness lasting about half an hour. She denies any history of fever, trauma, rash, burning micturition, urethral discharge, or diahorrea. She consulted the same practitioner outside and was diagnosed as cellulitis and given oral linezolid and clindamycin, but she showed no signs of improvement after one week, and her joint pain worsened. Thus she was admitted and was given injectables including vancomycin and piperacillin-tazobactam along with topical application of magnesium sulphate to reduce edema. She was referred to our institute, and a rheumatology opinion was sought regarding persisting joint pain.

**Figure 1:**
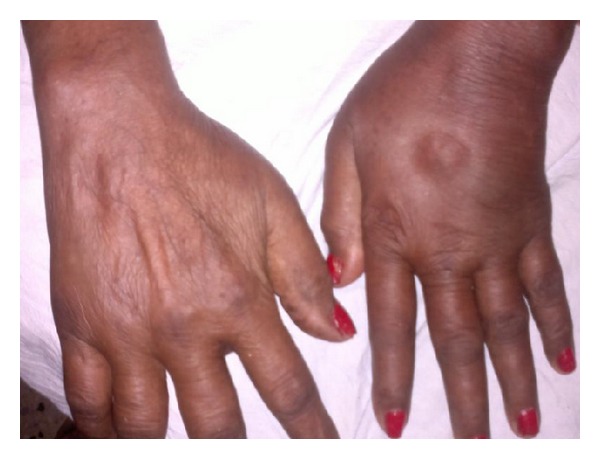
Showing clinical photograph of the patient.

**Figure 2:**
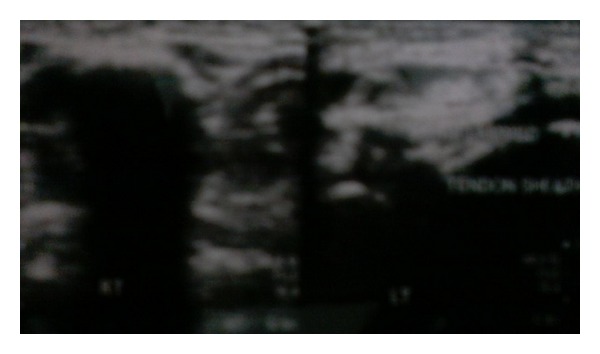
Color Doppler of limb showing inflamed tendon sheaths.

On examination, there was marked pitting edema of left hand extending up to wrist joint (Figure 1). It was not warm but extremely tender. There was limitation of movements of wrist and MCP joints, but there was no neurological deficit. Her vitals were within normal limits, and in rest systemic examination (including locomotor), no abnormality was detected. Laboratory investigations revealed hemoglobin = 9.4, total count = 6500, ESR = 44 mm, and CRP = 23.8. Both RA and anti-CCP were negative. X-ray of hands was normal without any evidence of erosions and fracture. Color Doppler sonography of left hand reveals extensor tenosynovitis of hands (Figure 2).

The diagnosis of unilateral RS3PE was made. Patient was given oral prednisolone (15 mg/day). The patient responded as pain starts subsiding within a week, and all symptoms were relieved within three weeks. She is in regular followup and is without any residual disability and deformity.

## 3. Discussion

RS3PE was considered a symmetrical entity until Pariser and Canoso [4] reported two cases of unilateral RS3PE. Both patients had neurological deficit, as case 1 has Erb's palsy, and case 2 was a known case of stroke with left hemiplegia. RS3PE occurred at the extremity that was spared. Later Finnell and Cuesta [5] reported asymmetrical involvement of lower limbs in his case series with greater involvement of right lower limb in case 2. Keenan et al. [6] later reported a case of right hemiplegic patient presenting with left sided RS3PE. Three more cases [7–9] including two left sided and one right sided were also reported. The majority of them were associated with neurological disturbances, and association with rheumatoid arthritis or any other autoimmune disease was not shown in any of them.

The mechanism which leads to genesis of RS3PE is still under dark, but a high prevalence of unilateral RS3PE in patients with neurological deficits (central as well as peripheral) raises the suspicion that local and neurological factors play a role in pathogenesis and progression of disease. Animal studies have shown that inflammatory and joint destructive response is decreased on the side of neurological impairment [10]. This is supported clinically by occurrence of unilateral disease on the side contralateral to side of neurological deficit. Role of vascular endothelial growth factor in pathogenesis of RS3PE is well established [11]. Animal models suggested that in neurological injuries there is downregulation of VEGF receptors [12]. Thus the neurological affected site may be resistant to development of edema. However, presence of unilateral disease in patients with autoimmune diseases is area still unexploited and needs more research which could establish the pathogenesis. 

Previously Özşahin et al. [13] reported a case of unilateral RS3PE that occurs in a patient of long-standing rheumatoid arthritis who was started on DMARDs but later left them, as his symptoms were not controlled. RS3PE developed three years after leaving the drugs. On the contrary, in our case symptoms were well controlled with DMARDs, and patient developed RS3PE when she was on medication. To the best of our knowledge, this is the first case of unilateral RS3PE in a patient of rheumatoid arthritis, while on drugs. This case also adds to clinical aspect according to which RS3PE is considered as a distinct clinical entity than rheumatoid arthritis as RS3PE occurred in a patient regularly taking DMARD.

To conclude, lack of specific diagnostic criteria and an asymmetrical unilateral pattern always poses diagnosis of RS3PE as a challenge. However, the diagnosis must be kept whenever a patients presents with acute onset polyarthritis with pitting edema on the back of extremity and a negative rheumatoid factor. Though the disease is rare in comparison to other common rheumatologic disorders as rheumatoid arthritis, it has an excellent prognosis. Misdiagnosis not only increases the duration of pain in the patient, but it also puts them on heavy cost of drugs.

## References

[1] McCarty DJ, O’Duffy JD, Pearson L, Hunter JB (1985). Remitting seronegative symmetrical synovitis with pitting edema. *The Journal of the American Medical Association*.

[2] Russell EB (2005). Remitting seronegative symmetrical synovitis with pitting edema syndrome: followup for neoplasia. *Journal of Rheumatology*.

[3] Olivé A, del Blanco J, Pons M, Vaquero M, Tena X (1997). The clinical spectrum of remitting seronegative symmetrical synovitis with pitting edema. *Journal of Rheumatology*.

[4] Pariser KM, Canoso JJ (1991). Remitting, seronegative (A) symmetrical synovitis with pitting edema—two cases of RS3PE syndrome. *Journal of Rheumatology*.

[5] Finnell JA, Cuesta IA (2000). Remitting seronegative symmetrical synovitis with pitting edema (RS3PE) syndrome: a review of the literature and a report of three cases. *Journal of Foot and Ankle Surgery*.

[6] Keenan RT, Hamalian GM, Pillinger MH (2009). RS3PE presenting in a unilateral pattern: case report and review of the literature. *Seminars in Arthritis and Rheumatism*.

[7] Olivieri I, Padula A, Favaro L, Oranges GS, Ferri S (1994). RS3PE syndrome with unilateral involvement. *Journal of Rheumatology*.

[8] Pierro A, Favaro L, Olivieri I, Amoresano C, Ferri S (1995). Another case of RS3PE with unilateral involvement. *Reumatismo*.

[9] Segerer S, Dietz-Laukemann P, Schattenkirchner M (1999). RS3PE-syndrome. *Zeitschrift fur Rheumatologie*.

[10] Courtright LJ, Kuzell WC (1965). Sparing effect of neurological deficit and trauma on the course of adjuvant arthritis in the rat. *Annals of the Rheumatic Diseases*.

[11] Arima K, Origuchi T, Tamai M (2005). RS3PE syndrome presenting as vascular endothelial growth factor associated disorder. *Annals of the Rheumatic Diseases*.

[12] Brockington A, Wharton SB, Fernando M (2006). Expression of vascular endothelial growth factor and its receptors in the central nervous system in amyotrophic lateral sclerosis. *Journal of Neuropathology and Experimental Neurology*.

[13] Özşahin M, Ataoğlu S, Turan H (2011). Unilateral RS3PE with young-onset rheumatoid arthritis. *Seminars in Arthritis and Rheumatism*.

